# Trends, gender, and racial disparities in patients with mortality due to paroxysmal tachycardia: A nationwide analysis from 1999–2020

**DOI:** 10.1371/journal.pone.0314715

**Published:** 2025-02-04

**Authors:** Aman Goyal, Humza Saeed, Saif Yamin, Abdullah, Wania Sultan, Muhammad Khubaib Arshad, Samia Aziz Sulaiman, Mah I. Kan Changez, Gauranga Mahalwar

**Affiliations:** 1 Department of Internal Medicine, Seth GS Medical College and KEM Hospital, Mumbai, India; 2 Department of Internal Medicine, Rawalpindi Medical University, Rawalpindi, Pakistan; 3 School of Medicine, University of Jordan, Amman, Jordan; 4 Department of Internal Medicine, Dow University of Health Sciences, Karachi, Pakistan; 5 Department of Cardiothoracic Surgery, Yale University, New Haven, Connecticut, United States of America; 6 Cleveland Clinic Foundation, Cleveland, Ohio, United States of America; Baruch Padeh Medical Center Poriya, ISRAEL

## Abstract

**Background:**

Paroxysmal tachycardia encompasses various heart rhythm disorders that cause rapid heart rates. Its episodic occurrence makes it difficult to identify and measure its prevalence and trends in the population. Additionally, there is limited data on disparities and trends in mortality due to paroxysmal tachycardia, which is essential for assessing current medical approaches and identifying at-risk populations.

**Methods:**

Our study examined death certificates from 1999 to 2020 using the CDC WONDER Database to identify deaths caused by paroxysmal tachycardia in individuals aged 25 and older, using the ICD-10 code I47. Age-adjusted mortality rates (AAMRs) and annual percent changes (APC) were calculated by year, gender, age group, race/ethnicity, geographic location, and urbanization status. Trends in AAMRs were analyzed using the Joinpoint Regression Program to identify significant changes and inflection points in mortality trends throughout the study period.

**Results:**

Between 1999 and 2020, 155,320 deaths were reported in patients with paroxysmal tachycardia. Overall, AAMR decreased from 4.8 to 3.7 per 100,000 population between 1999 and 2020, despite showing a significant increase from 2014 to 2020 (APC: 4.33; 95% CI: 3.53 to 5.56). Men had consistently higher AAMRs than women (4.7 vs. 2.2). Furthermore, we found that AAMRs were highest among Non-Hispanic (NH) Black or African Americans and lowest in NH Asian or Pacific Islanders (4 vs. 1.9). Nonmetropolitan areas had higher AAMRs than metropolitan areas (3.6 vs. 3.2).

**Conclusions:**

Our analysis showed a significant decrease in mortality from paroxysmal tachycardia since 1999, although there has been a slight increase in recent years. However, disparities remain, with higher AAMRs among men, NH Black or African Americans, and residents of non-metropolitan areas. These findings call for immediate public health actions to curb the rising trends and reduce potential disparities.

## Introduction

Paroxysmal tachycardia, also known as episodic tachycardia, encompasses a subset of abnormal heart rhythms, including re-entry ventricular arrhythmia, supraventricular tachycardia (SVT), and ventricular tachycardia (VT). Mismanagement of any of these conditions can have lethal consequences. Among them, re-entrant arrhythmia has been associated with relatively low mortality rates. However, it still has been found to pose long-term health risks, including stroke and thromboembolism in specific subtypes [1–3].

SVT has been linked to a higher incidence in structural heart disease (SHD), occurring in up to 32% of the SHD population [3]. Therefore, due to its association with SHD, it is considered the most common arrhythmia in children, affecting around 1 in 250 otherwise healthy children [2, 3]. Moreover, a large multicenter study revealed that 82% of deaths of SHD-patients occurred due to SVTs [4]. Simultaneously, VT posed a substantial risk of mortality and ranked as the fifth most frequent cause of emergency department visits among patients between 65 to 84 years of age [5]. Moreover, it was also found that 30% to 75% of out-of-hospital cardiac arrests were attributed to VTs, adding to its dangerous nature [6].

Despite the relatively favorable patient outcomes associated with paroxysmal tachycardia, tachyarrhythmias significantly affect healthcare systems and survival rates, and have been closely tied to an increased risk of mortality, comorbidities, and complex cardiac defects [7]. As far as medical management is concerned, although implantable cardioverter defibrillators have demonstrated efficacy in reducing VT-related deaths, it is revealed that they unfortunately do not mitigate the risk of VT recurrences [8].

Hence, it is imperative to explore the demographic and regional mortality patterns among US adults with paroxysmal tachycardia. By exploring the trends, gender, and racial disparities in mortality due to paroxysmal tachycardia, our study aims to identify potentially vulnerable populations to guide future research and clinical practice to improve prevention strategies and develop smarter frameworks to reduce complications associated with this condition. Additionally, our research will contribute to better healthcare outcomes and improved quality of life for affected individuals.

## Methods

### Study setting and population

We obtained mortality data on June 15, 2024, from the Centers for Disease Control and Prevention’s WONDER database which provides comprehensive epidemiological information based on death certificates in the US [9]. Our investigation focused on mortality rates among adult individuals (aged 25 years or older) affected by Paroxysmal Tachycardia between 1999 and 2020. Several previously published studies have employed this methodology using similar year ranges [10–13]. Using the International Statistical Classification of Diseases and Related Health Problems, 10th Revision [14], we assigned code I47 to Paroxysmal Tachycardia, which included 147.0, 147.1, 147.2, and 147.9 for Re-entry ventricular arrhythmia, Supraventricular tachycardia, Ventricular tachycardia, and unspecified Paroxysmal tachycardia, respectively. Specifically, we utilized the Multiple Cause-of-Death Public Use Record database to identify instances where paroxysmal tachycardia was recorded either as the underlying cause or as a contributing cause of death on death certificates across the US [15]. Institutional review board approval was not required, as we utilized a de-identified government-provided public-use dataset in accordance with Strengthening the Reporting of Observational Studies in Epidemiology (STROBE) guidelines [16].

### Data abstraction

Our investigation analyzed demographic variables, including population size, age distribution, gender composition, racial and ethnic background, geographic location, urbanization level, and place of death. The locations of death spanned diverse settings, including inpatient facilities, outpatient clinics, emergency rooms, sudden death cases, residences, hospice/nursing homes, long-term care facilities, and instances where the location remained unspecified. We meticulously defined racial and ethnic categories, encompassing Hispanic (Latino), Non-Hispanic (NH) White, NH Black/African American, NH American Indian/Alaskan Native, and NH Asian. These classifications align with those previously used in analyses from the CDC WONDER database and are based on data reported on death certificates in compliance with the US Office of Budget and Management Guidelines [17].

In accordance with the age-wise analysis, we categorized patients into ten-year intervals, distinguishing young adults (25–44 years), middle-aged adults (45–64 years), and older individuals (65–85+ years) in accordance with the previously published studies [18, 19]. We utilized the Urban-Rural Classification Scheme from the National Center for Health Statistics to classify our study population geographically. Urban areas housed populations of 50,000 or more, while Rural areas included locales with fewer than 50,000 residents. Furthermore, we divided the United States into four regions based on the US Census Bureau’s classification: Northeast, Midwest, South, and West [20].

### Statistical analysis

We analyzed patterns related to gender, race, age, urbanization, and census regions by calculating both crude and age-adjusted mortality rates (AAMR) per 100,000 individuals for paroxysmal tachycardia. The 2000 US population served as the reference standard for AAMR calculations [21]. To evaluate changes in mortality rates over time, we used the Joinpoint Regression Program (Version 5.0.2, National Cancer Institute) [22].

#### Parameter settings of the Joinpoint regression model

Temporal trends in AAMR were analyzed using log-linear regression models. Joinpoint regression was applied to detect shifts or inflection points in the temporal trends of AAMR for paroxysmal tachycardia from 1999 to 2020, in line with established methodological guidelines. For datasets with 17 to 21 time points, these guidelines suggest identifying a maximum of three inflection points. Since our study spanned 22 years, the software was configured to detect up to four joinpoints where significant variations in trends occurred. Nonetheless, fewer inflection points could be selected if the greatest variation between trends was achieved with fewer joinpoints. The Grid Search method (2, 2, 0), along with permutation testing and parametric methods, was used to calculate the annual percent change (APC) and 95% confidence intervals (CIs). An APC was classified as increasing or decreasing if the slope of the change in mortality was significantly different from zero, using a 2-tailed t-test. Statistical significance was defined as P≤0.05.

## Results

Between 1999 and 2020, there were 155,320 deaths in which paroxysmal tachycardia was recorded as either the underlying or a contributing cause (S1 Table). The place of death was documented for 152,390 of these cases, with 75.7% occurring in medical facilities, 13.3% at the decedents’ homes, 7.7% in nursing homes or long-term care facilities, and 1.4% in hospices (S2 Table).

### Overall trends in mortality

**Fig 1:**
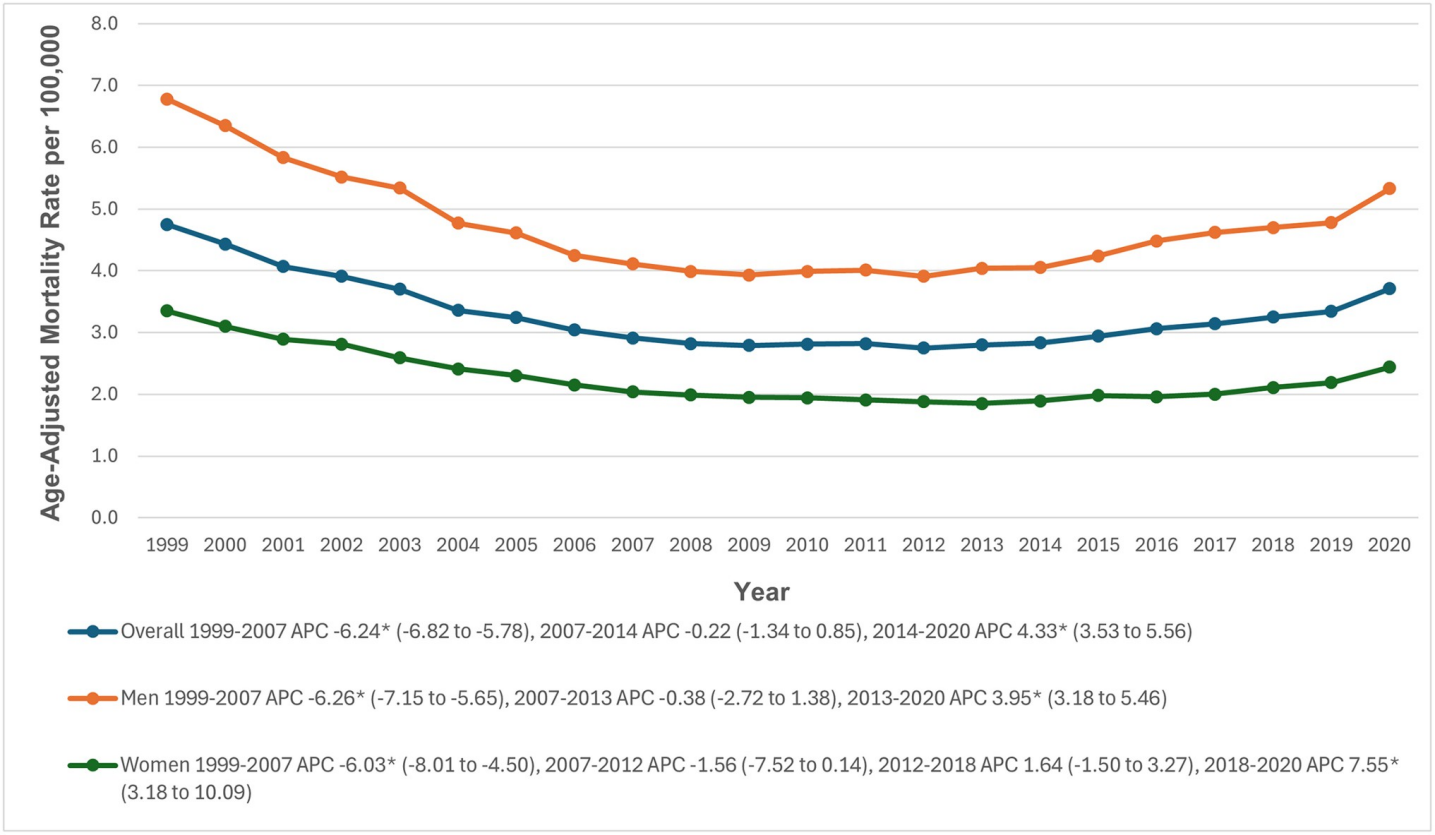
Overall and sex-stratified paroxysmal tachycardia-related age-adjusted mortality rates per 100,000 in adults in the United States, 1999 to 2020. * Indicates that the annual percentage change (APC) is significantly different from zero at α = 0.05. AAMR = age-adjusted mortality rate.

The AAMR was 4.8 (95% CI: 4.7 to 4.9) in 1999 and decreased to 3.7 (95% CI: 3.2 to 3.3) in 2020. The overall AAMR showed a significant decline from 1999 to 2007 (APC: -6.24; 95% CI: -6.82 to -5.78), followed by a minor decrease from 2007 to 2014 (APC: -0.22; 95% CI: -1.34 to 0.85). This trend then reversed, with a significant increase observed from 2014 to 2020 (APC: 4.33; 95% CI: 3.53 to 5.56) (Fig 1, S3 and S4 Tables).

### Gender stratification

During the study period, men’s AAMRs were higher than women’s (Men: 4.7; 95% CI: 4.6 to 4.7; Women: 2.2; 95% CI: 2.2 to 2.2). In 1999, the average AAMR for men was 6.8 (95% CI: 6.6 to 7.0), which decreased significantly to 4.1 (95% CI: 4.0 to 4.2) in 2007 (APC: -6.26; 95% CI: -7.15 to -5.65). The rate remained stable from 2007 to 2013 (APC: -0.38; 95% CI: -2.72 to 1.38), followed by a significant increase to 5.3 in 2020 (APC: 3.95; 95% CI: 3.18 to 5.46). For women, the AAMR was 3.4 (95% CI: 3.2 to 3.5) in 1999, significantly dropping to 2.0 (95% CI: 2.0 to 2.1) in 2007 (APC: -6.03; 95% CI: -8.01 to -4.50), and further declining to 1.9 in 2012 (APC: -1.56; 95% CI: -7.52 to 0.14). It then rose to 2.1 in 2018 (APC: 1.64; 95% CI: -1.50 to 3.27) and increased further to 2.4 in 2020 (APC: 7.55; 95% CI: 3.18 to 10.09) (Fig 1, S3 and S4 Tables).

### Stratification by age groups

**Fig 2:**
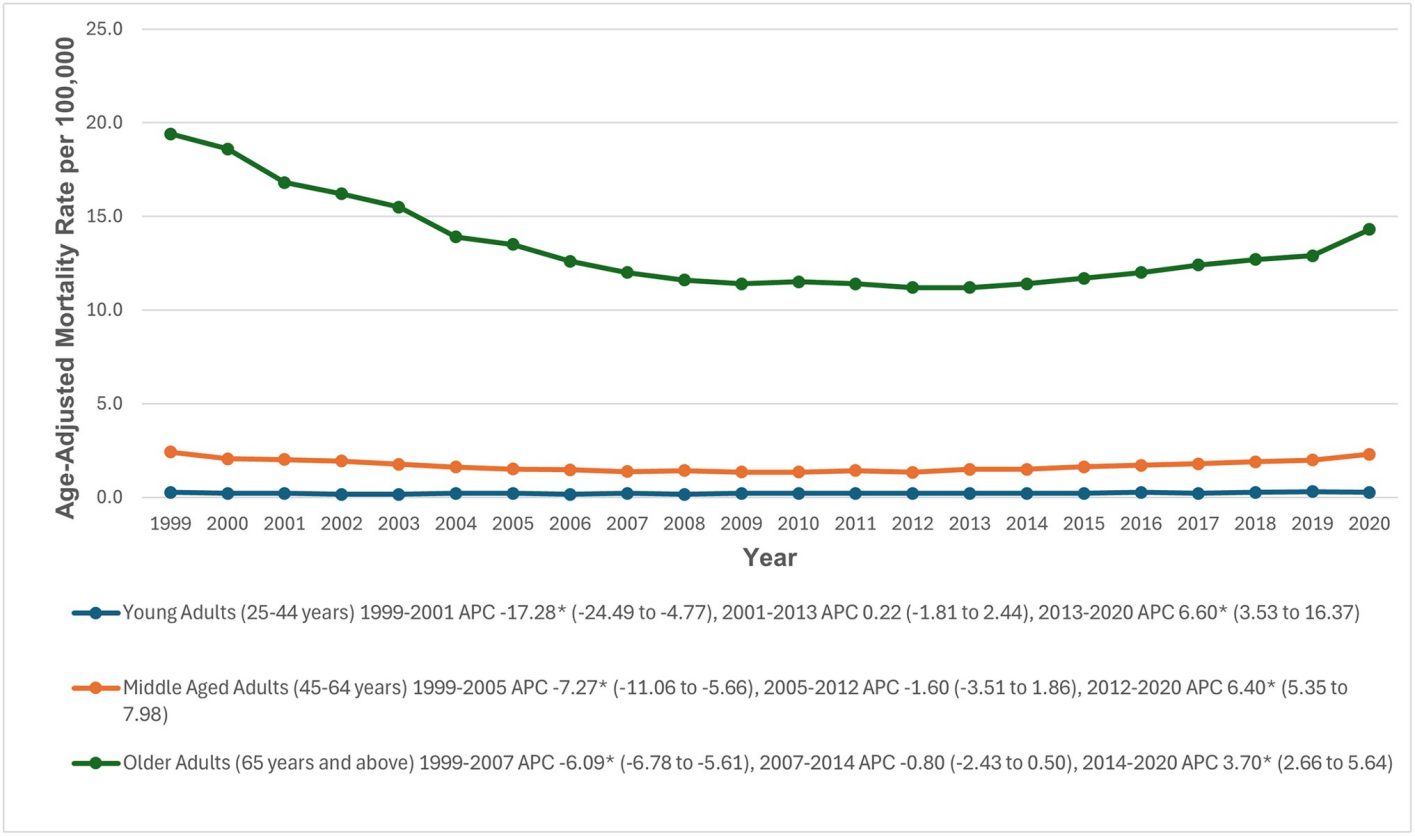
Paroxysmal tachycardia-related age-adjusted mortality rates per 100,000, stratified by age groups in adults in the United States, 1999 to 2020. * Indicates that the annual percentage change (APC) is significantly different from zero at α = 0.05. AAMR = age-adjusted mortality rate.

When stratified by age groups, older adults had the highest AAMRs (13.3; 95% CI: 13.2 to 13.3), followed by middle-aged adults (1.7; 95% CI: 1.6 to 1.7), and young adults (0.2; 95% CI: 0.2 to 0.2). Among young adults, AAMRs significantly decreased from 1999 to 2001 (APC: -17.28; 95% CI: -24.49 to -4.77). A similar declining trend was observed in middle-aged and older adults from 1999 to 2005 (APC: -7.27; 95% CI: -11.06 to -5.66) and from 1999 to 2007 (APC: -6.09; 95% CI: -6.78 to -5.61), respectively. From 2001 to 2013, the AAMR among young adults remained stable (APC: 0.22; 95% CI: -1.81 to 2.44), followed by a significant increase through 2020 (APC: 6.60; 95% CI: 3.53 to 16.37). AAMRs decreased from 2005 to 2012 in middle-aged adults (APC: -1.60; 95% CI: -3.51 to 1.86) and from 2007 to 2014 in older adults (APC: -0.80; 95% CI: -2.43 to 0.50). This decline was followed by a significant increase through 2020 in both middle-aged (APC: 6.40; 95% CI: 5.35 to 7.98) and older adults (APC: 3.70; 95% CI: 2.66 to 5.64) (Fig 2, S3 and S5 Tables).

### Racial and ethnic stratification

**Fig 3:**
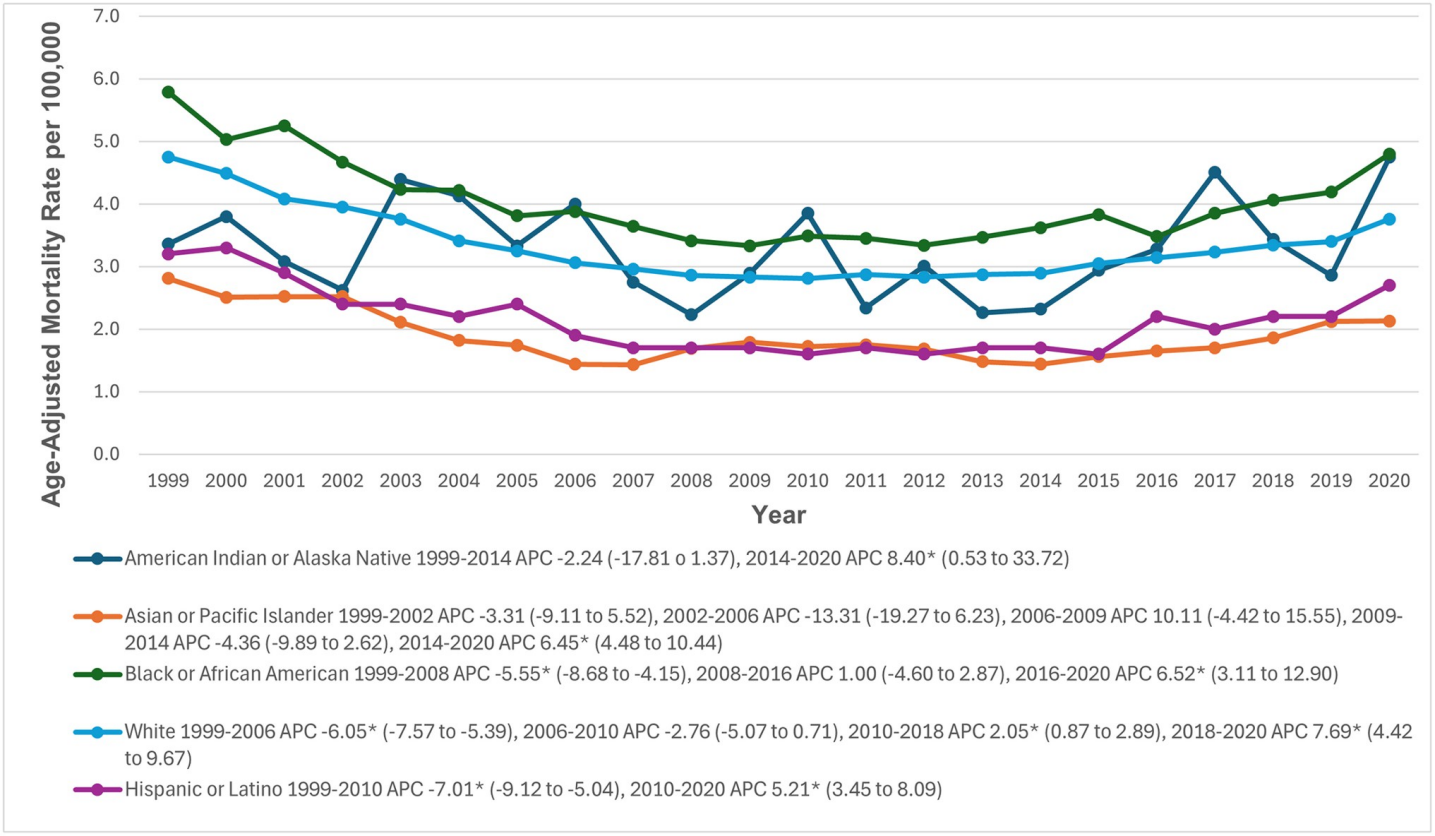
Paroxysmal tachycardia-related age-adjusted mortality rates per 100,000, stratified by race in adults in the United States, 1999 to 2020. * Indicates that the annual percentage change (APC) is significantly different from zero at α = 0.05. AAMR = age-adjusted mortality rate.

When stratified by race and ethnicity, AAMRs were highest among NH Black or African American patients (4.0; 95% CI: 3.9 to 4.1), followed by NH American Indian or Alaska Native (3.3; 95% CI: 3.0 to 3.5), NH White (3.3; 95% CI: 3.3 to 3.4), Hispanic (2.1; 95% CI: 2.1 to 2.2), and NH Asian or Pacific Islander populations (1.9; 95% CI: 1.8 to 1.9). For NH American Indians or Alaska Natives, AAMRs decreased from 1999 to 2014 (APC: -2.24; 95% CI: -17.81 to 1.37), followed by a significant increase to 2020 (APC: 8.40; 95% CI: 0.53 to 33.72). Similarly, AAMRs for Hispanics significantly decreased from 1999 to 2010 (APC: -7.01; 95% CI: -9.12 to -5.04) and then increased significantly until 2020 (APC: 5.21; 95% CI: 3.45 to 8.09). Among NH Black or African Americans, AAMRs significantly decreased from 1999 to 2008 (APC: -5.55; 95% CI: -8.68 to -4.15), followed by a minor increase from 2008 to 2016 (APC: 1.00; 95% CI: -4.60 to 2.87), and a significant increase to 2020 (APC: 6.52; 95% CI: 3.11 to 12.90). For NH Asians, AAMRs decreased between 1999 and 2002 (APC: -3.31; 95% CI: -9.11 to 5.52) and again from 2002 to 2006 (APC: -13.31; 95% CI: -19.27 to 6.23). This was followed by an increase from 2006 to 2009 (APC: 10.11; 95% CI: -4.42 to 15.55), a decrease from 2009 to 2014 (APC: -4.36; 95% CI: -9.89 to 2.62), and a significant increase until 2020 (APC: 6.45; 95% CI: 4.48 to 10.44). For NH Whites, AAMRs decreased significantly from 1999 to 2006 (APC: -6.05; 95% CI: -7.57 to -5.39) and declined further from 2006 to 2010 (APC: -2.76; 95% CI: -5.07 to 0.71). This was followed by a significant increase from 2010 to 2018 (APC: 2.05; 95% CI: 0.87 to 2.89) and from 2018 to 2020 (APC: 7.69; 95% CI: 4.42 to 9.67) (Fig 3, S3 and S6 Tables).

### State-wise distribution

**Fig 4:**
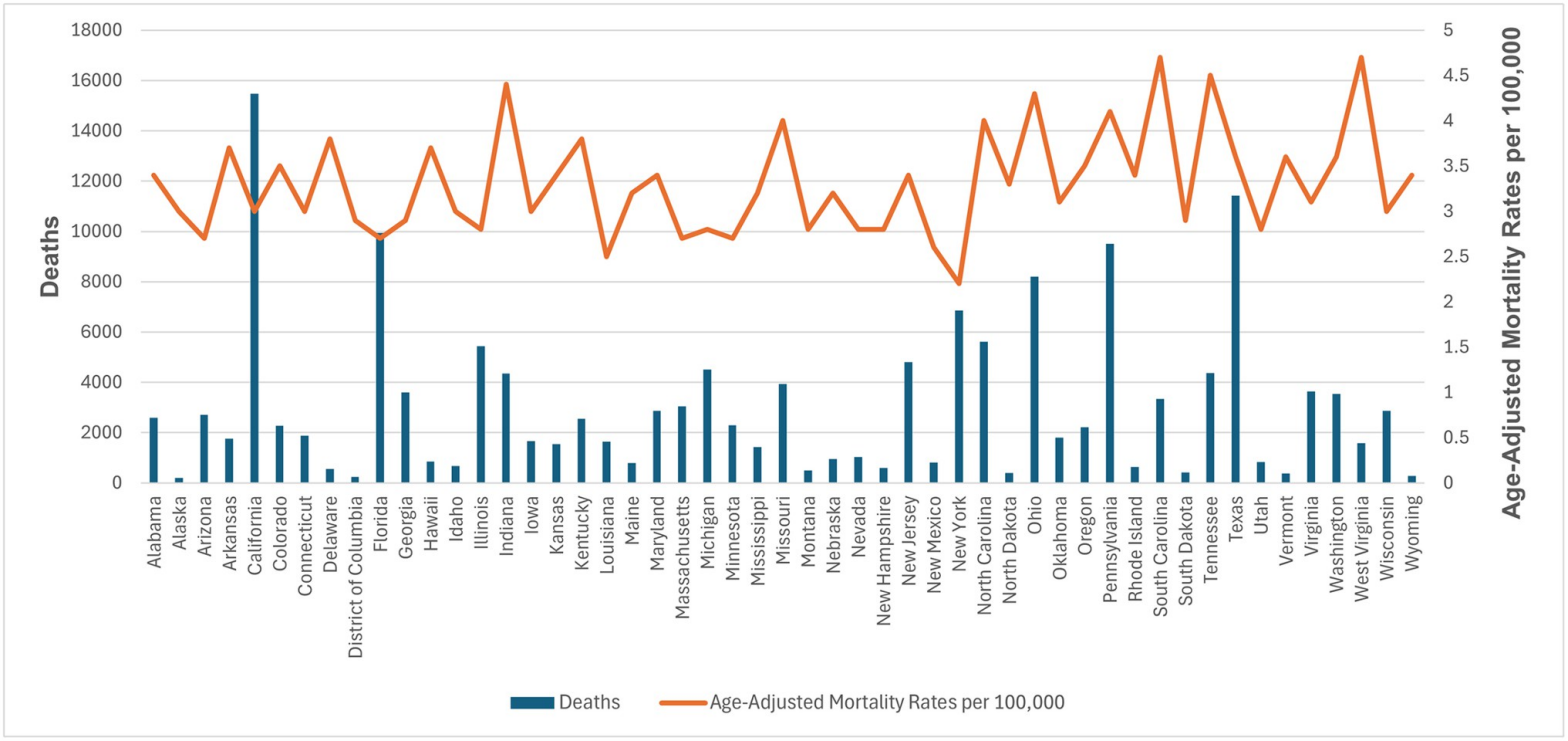
Paroxysmal tachycardia-related deaths and age-adjusted mortality rates per 100,000, stratified by state in adults in the United States, 1999 to 2020.

AAMR values varied significantly by state, ranging from 2.2 (95% CI: 2.1 to 2.2) in New York to 4.7 (95% CI: 4.5 to 4.9) in West Virginia. States in the top 90th percentile (Pennsylvania, Ohio, Indiana, Tennessee, South Carolina, West Virginia) had AAMRs about twice as high as those in the bottom 10th percentile (New York, Louisiana, New Mexico, Florida, Arizona, Massachusetts, Minnesota) (Fig 4, S7 Table).

### Census region

**Fig 5:**
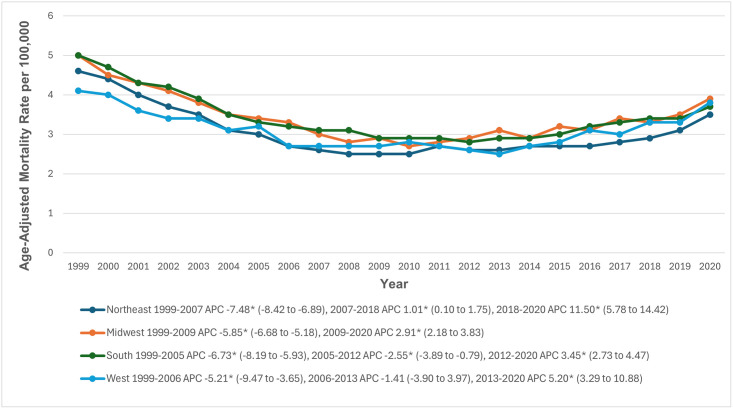
Paroxysmal tachycardia-related age-adjusted mortality rates per 100,000, stratified by census regions in adults in the United States, 1999 to 2020. * Indicates that the annual percentage change (APC) is significantly different from zero at α = 0.05. AAMR = age-adjusted mortality rate.

During the study period, AAMRs varied across the census regions, with the highest rates observed in the Midwestern and Southern regions (3.4; 95% CI: 3.4 to 3.4), followed by the Western (3.1; 95% CI: 3.0 to 3.1) and Northeastern (3.0; 95% CI: 3.0 to 3.1) regions. In the Midwestern region, AAMRs decreased significantly from 1999 to 2009 (APC: -5.85; 95% CI: -6.68 to -5.18), then increased significantly through 2020 (APC: 2.91; 95% CI: 2.18 to 3.83). In the Southern region, AAMRs decreased significantly from 1999 to 2005 (APC: -6.73; 95% CI: -8.19 to -5.93), followed by another significant decrease from 2005 to 2012 (APC: -2.55; 95% CI: -3.89 to -0.79), and then a significant increase through 2020 (APC: 3.45; 95% CI: 2.73 to 4.47). The Western region showed a significant decrease from 1999 to 2006 (APC: -5.21; 95% CI: -9.47 to -3.65), followed by a smaller decrease from 2006 to 2013 (APC: -1.41; 95% CI: -3.90 to 3.97), and a subsequent significant increase through 2020 (APC: 5.20; 95% CI: 3.29 to 10.88). In the Northeastern region, AAMRs significantly decreased from 1999 to 2007 (APC: -7.48; 95% CI: -8.42 to -6.89), followed by a significant increase from 2007 to 2018 (APC: 1.01; 95% CI: 0.10 to 1.75) and a further increase from 2018 to 2020 (APC: 11.50; 95% CI: 5.78 to 14.42) (Fig 5, S3 and S8 Tables).

### Urbanization

**Fig 6:**
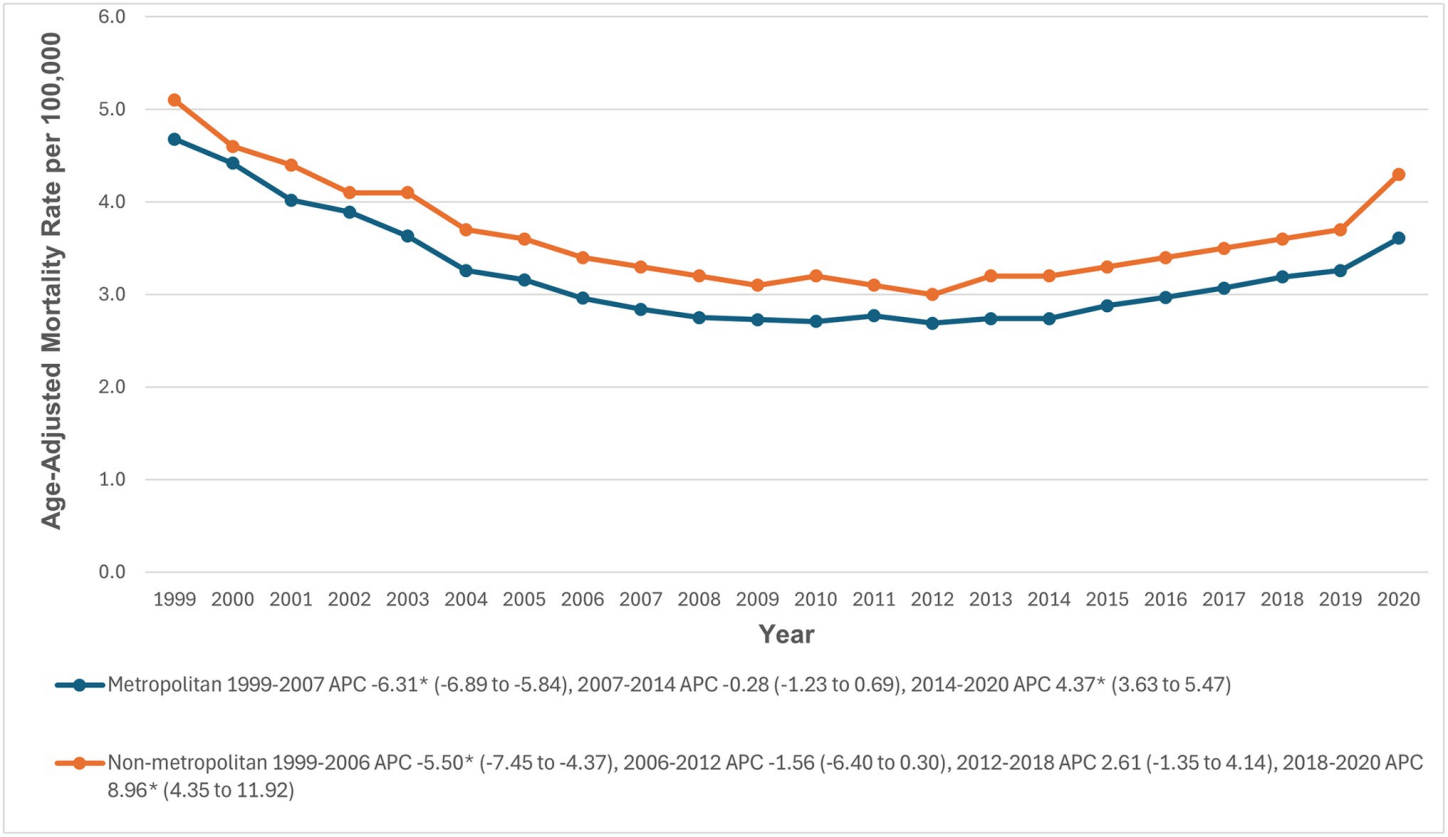
Paroxysmal tachycardia-related age-adjusted mortality rates per 100,000 in adults in the metropolitan and non-metropolitan areas in the United States, 1999 to 2020. * Indicates that the APC is significantly different from zero at α = 0.05. AAMR = age-adjusted mortality rate.

Throughout the study period, non-metropolitan areas had higher AAMRs for paroxysmal tachycardia than metropolitan areas, with overall AAMRs of 3.6 (95% CI: 3.6 to 3.6) and 3.2 (95% CI: 3.2 to 3.2), respectively. In the metropolitan group, AAMRs significantly decreased from 1999 to 2007 (APC: -6.31; 95% CI: -6.89 to -5.84), followed by a minor decrease from 2007 to 2014 (APC: -0.28; 95% CI: -1.23 to 0.69), and then a significant increase through 2020 (APC: 4.37; 95% CI: 3.63 to 5.47). Among the non-metropolitan group, AAMRs significantly decreased from 1999 to 2006 (APC: -5.50; 95% CI: -7.45 to -4.37), followed by a gradual decrease from 2006 to 2012 (APC: -1.56; 95% CI: -6.40 to 0.30). The AAMRs then increased from 2012 to 2018 (APC: 2.61; 95% CI: -1.35 to 4.14), followed by a significant increase through 2020 (APC: 8.96; 95% CI: 4.35 to 11.92) (Fig 6, S3 and S9 Tables).

## Discussion

The current study reveals trends, gender, age, racial, and geographical differences in mortality due to paroxysmal tachycardia. Up to the authors’ best knowledge, this is the first study to investigate these trends from 1999 to 2020 in the US population. Data was obtained from the Centers for Disease Control and Prevention’s WONDER database. The data comprised 155,320 deaths caused by paroxysmal tachycardia from 1999 to 2020. During the period of 1999 to 2008, there was a steady decline in mortality due to paroxysmal tachycardia. However, it was followed by a steady incline in mortality rate, reaching 10008 deaths in 2020, which is higher than deaths recorded in 1999, which accounted for 8387 cases. This finding could be attributed to the fact that cardiac arrhythmias has been documented as a potential complication of COVID-19 [23].

The trends in gender and age differences noted in our study signal a critical area for further investigation and intervention. Men had higher AAMRs as compared to women. This variation may result from differences in the risk of ischemic heart disease and variations in the susceptibility of different genders to arrhythmias [24]. Gender differences have been associated with certain claims relating to hormones, where it has been documented that testosterone lowers the calcium release facilitated by ryanodine receptors and the sodium/calcium exchange current [24]. Due to the opposite action of estrogen, women may be more susceptible to provocative activity [25, 26]. On the contrary, estrogen is also known to be cardioprotective, as indicated by the decrease of cardiac cell death in cardiomyopathies while testosterone stimulates the production of inflammatory substances [27].

Regarding age groups, older adults had the highest AAMRs due to paroxysmal tachycardia compared to the middle and young adult age groups, which could be attributed to older adults having a higher number of comorbidities placing them at increased risk [28]. Notably, there has been an increase in AAMR among all age groups in the last decade. This may be partly attributed to the COVID-19 pandemic, as arrhythmias are potential complications, and also to increased accessibility to healthcare and improved technology, which facilitates diagnosis [29–31].

In the context of racial differences, the most significant mortality due to paroxysmal tachycardia was noted in the NH Black or African American group, followed by the NH Indian American racial population. Racial differences are most likely due to socioeconomic and environmental factors [32]. This gap may be linked to recognized discrepancies in healthcare access and resources encountered by these minority communities [33]. Such could include income and insurance differences, decreased access to healthcare, and the prevalence of unmanaged comorbidities, which could all act as risk factors for paroxysmal tachycardia and arrhythmias. Future research must therefore focus on exploring socioeconomic factors which could contribute to potential disparities. Moreover, this is also reinforced by previous evidence, which highlights greater COVID-19 infection rates and deaths within these groups [34].

Our study revealed significant geographic variations in AAMRs for paroxysmal tachycardia across the United States, with the highest rates observed in the Midwestern and Southern regions. These areas consistently showed higher mortality compared to the Western and Northeastern regions, likely due to differences in healthcare access, socioeconomic conditions, and the prevalence of cardiovascular risk factors such as hypertension and diabetes [35, 36]. State-level analysis further highlights these disparities, with states like West Virginia, Ohio, and Pennsylvania exhibiting notably higher AAMRs compared to states like New York and Massachusetts. This trend may be explained, in part, by the larger rural populations within these high-AAMR states, where access to specialized cardiac care is often limited, and chronic conditions are more prevalent [36].

These geographic patterns align with our findings on urbanization trends, where non-metropolitan areas exhibited higher AAMRs than metropolitan regions. Limited healthcare access in rural areas may have widened the mortality gap between urban and rural populations, particularly over the last decade [37]. This suggests that the disparities observed on a regional level are reflected locally, with non-metropolitan areas facing similar healthcare challenges. While telehealth may not be a solution for every patient, its use, as demonstrated in a study by Soliman et al., could help bridge the healthcare access gap by managing primary and secondary cardiovascular diseases in larger, underserved populations [38]. Telehealth platforms can be particularly beneficial for following up with patients with comorbidities and reducing complications associated with delayed care.

**Future prospects**. Overall, this study reveals a significant mortality rate associated with paroxysmal tachycardia in the United States. By addressing possible contributory variables such as pandemic effects and racial and ethnic inequalities, healthcare providers and policymakers may devise comprehensive solutions to combat this concerning trend. Education and information should not only focus on identifying risk factors but also on modifying the socio-cultural contexts that lead to the same risk factors. In this setting, tight partnerships between policymakers, healthcare providers, and doctors are also crucial for ensuring a favorable cost-effectiveness of health spending [39].

### Limitations

Despite the robustness of our data and analytical methods, this study has several limitations that should be acknowledged. Firstly, the use of ICD-10 codes and reliance on death certificates from the CDC WONDER database may introduce inaccuracies, such as misclassification or underreporting of paroxysmal tachycardia as a cause or contributing factor in mortality records. Secondly, while our analysis focused on broad demographic trends, it did not adjust for potential confounding factors such as body weight, diet, lifestyle behaviors (including physical activity), smoking status, or the presence of other underlying diseases. These factors could significantly influence the risk and mortality associated with paroxysmal tachycardia, and their exclusion limits the depth of our analysis. Thirdly, the absence of detailed information regarding medical treatments or management approaches limits our ability to evaluate how different therapeutic strategies might have influenced mortality trends. Additionally, differences in diagnostic practices and healthcare resources between rural and urban healthcare facilities could affect the completeness and accuracy of recorded mortality data. Lastly, the reliance on aggregate-level data limits our ability to draw causal inferences between observed mortality trends and specific healthcare interventions or policy shifts over time. Future research should aim to integrate more comprehensive clinical and socioeconomic data to better understand the factors influencing paroxysmal tachycardia-related mortality.

## Conclusions

Overall, our findings revealed that, despite a decrease in the rate of paroxysmal tachycardia-related mortality in the United States between 1999 and 2020, there is a rising mortality rate among males and NH Black or African Americans, particularly in non-metropolitan areas. This highlights that arrhythmia is becoming more prevalent in these populations. Furthermore, the increased rate in paroxysmal tachycardia-related mortality last year shows the need for additional research into etiology, which would aid in the implementation of tailored therapies to improve care in a specific group. Despite significant advances in treatment and secondary prevention approaches, this study emphasizes demographic differences. These findings necessitate immediate public health actions to reduce these potential disparities.

## Supporting information

S1 Table Paroxysmal tachycardia-related mortality, stratified by sex and race in adults in the United States, 1999 to 2020. (DOCX)

S2 Table Paroxysmal tachycardia-related mortality, stratified by place of death in adults in the United States, 1999 to 2020. N/A = not available (unreliable or suppressed).
(DOCX)

S3 Table Annual percent change (APC) of paroxysmal tachycardia–related age-adjusted mortality rates per 100,000 in adults in the United States, 1999 to 2020. APC = Annual percent change; NH = non-Hispanic.
(DOCX)

S4 Table Overall and sex‐stratified paroxysmal tachycardia–related age-adjusted mortality rates per 100,000 in adults in the United States from 1999 to 2020. (DOCX)

S5 Table Paroxysmal tachycardia-related mortality, stratified by age groups in adults in the United States, 1999 to 2020. Young Adult = 25–44 years; Middle Aged Adults = 45–64 years; Older Adults = 65 years and above.
(DOCX)

S6 Table Paroxysmal tachycardia-related age-adjusted mortality rates per 100,000 stratified by race in adults in the United States from 1999 to 2020. NH = non-Hispanic.
(DOCX)

S7 Table Paroxysmal tachycardia–related age-adjusted mortality rates per 100,000, stratified by state in adults in the United States, 1999 to 2020. (DOCX)

S8 Table Paroxysmal tachycardia related age-adjusted mortality rate per 100,000 stratified by census region in adults in the United States 1999–2020. (DOCX)

S9 Table Overall paroxysmal tachycardia–related age-adjusted mortality rates per 100,000 in adults in the metropolitan and non-metropolitan areas in the United States, 1999 to 2020. (DOCX)

S1 Graphical abstract (TIF)

## Abbreviations

AAMRs: Age-adjusted mortality rates
APC: Annual percentage change
NH: Non-Hispanic
SHD: structural heart disease
SVT: Supraventricular tachycardia
VT: ventricular tachycardia
