# Expression of Concern: Trends, gender, and racial disparities in patients with mortality due to paroxysmal tachycardia: A nationwide analysis from 1999–2020

**DOI:** 10.1371/journal.pone.0346401

**Published:** 2026-04-06

**Authors:** The PLOS One Editors

The *PLOS One* Editors issue this Expression of Concern to alert readers that this article [1] was identified as one of a series of submissions for which we have concerns about authorship and adherence to the journal’s first and second publication criteria [2]. We regret that these issues were not addressed prior to the article’s publication.
