# Supplementary material for: Trends, gender, and racial disparities in patients with mortality due to paroxysmal tachycardia: A nationwide analysis from 1999–2020
Source: PLoS One. 2025 Feb 4;20(2):e0314715. doi: 10.1371/journal.pone.0314715 (PMC11793763; doi:10.1371/journal.pone.0314715)
Supplement: S1 Table — (DOCX) [file pone.0314715.s001.docx]

**S1 Table.** Paroxysmal Tachycardia-related Mortality, Stratified by Sex and Race in Adults in the United States, 1999 to 2020

| Deaths | | | | | | | | | |
| --- | --- | --- | --- | --- | --- | --- | --- | --- | --- |
| Year | **Overall** | **Women** | **Men** | **NH White** | **NH Black or African American** | **NH Asian or Pacific Islander** | **NH American Indian or Alaska Native** | **Hispanic or Latino** | **Population** |
| 1999 | 8387 | 3546 | 4841 | 7062 | 887 | 118 | 26 | 275 | 180408769 |
| 2000 | 7933 | 3340 | 4593 | 6712 | 773 | 107 | 30 | 298 | 181984640 |
| 2001 | 7416 | 3128 | 4288 | 6138 | 826 | 115 | 31 | 289 | 184305128 |
| 2002 | 7227 | 3085 | 4142 | 6049 | 755 | 130 | 25 | 247 | 186208028 |
| 2003 | 6982 | 2891 | 4091 | 5855 | 692 | 117 | 38 | 273 | 188090429 |
| 2004 | 6398 | 2697 | 3701 | 5281 | 714 | 104 | 35 | 257 | 190205384 |
| 2005 | 6283 | 2624 | 3659 | 5192 | 654 | 105 | 32 | 292 | 192551384 |
| 2006 | 5988 | 2514 | 3474 | 4909 | 701 | 88 | 37 | 240 | 195019359 |
| 2007 | 5859 | 2430 | 3429 | 4842 | 667 | 95 | 25 | 224 | 197403777 |
| 2008 | 5811 | 2393 | 3418 | 4776 | 642 | 117 | 23 | 242 | 199795090 |
| 2009 | 5837 | 2375 | 3462 | 4772 | 639 | 129 | 32 | 258 | 202107016 |
| 2010 | 5944 | 2399 | 3545 | 4820 | 683 | 130 | 41 | 258 | 203891983 |
| 2011 | 6161 | 2438 | 3723 | 4998 | 698 | 145 | 28 | 286 | 206592936 |
| 2012 | 6183 | 2434 | 3749 | 4992 | 709 | 151 | 35 | 288 | 208826037 |
| 2013 | 6451 | 2454 | 3997 | 5176 | 766 | 142 | 34 | 318 | 211085314 |
| 2014 | 6650 | 2529 | 4121 | 5289 | 817 | 145 | 33 | 352 | 213809280 |
| 2015 | 7118 | 2716 | 4402 | 5638 | 898 | 166 | 41 | 347 | 216553817 |
| 2016 | 7510 | 2726 | 4784 | 5937 | 836 | 189 | 47 | 488 | 218641417 |
| 2017 | 7986 | 2875 | 5111 | 6238 | 977 | 203 | 69 | 482 | 221447331 |
| 2018 | 8409 | 3065 | 5344 | 6503 | 1036 | 236 | 55 | 557 | 223311190 |
| 2019 | 8779 | 3241 | 5538 | 6733 | 1130 | 282 | 53 | 570 | 224981167 |
| 2020 | 10008 | 3666 | 6342 | 7567 | 1314 | 301 | 82 | 725 | 226635013 |
| Total | 155320 | 61566 | 93754 | 125479 | 17814 | 3315 | 852 | 7566 | 4473854489 |

NH, non-Hispanic.
