# Supplementary material for: Trends, gender, and racial disparities in patients with mortality due to paroxysmal tachycardia: A nationwide analysis from 1999–2020
Source: PLoS One. 2025 Feb 4;20(2):e0314715. doi: 10.1371/journal.pone.0314715 (PMC11793763; doi:10.1371/journal.pone.0314715)
Supplement: S2 Table — N/A = not available (unreliable or suppressed). (DOCX) [file pone.0314715.s002.docx]

**S2 Table.** Paroxysmal Tachycardia-related Mortality, Stratified by Place of Death in Adults in the United States, 1999 to 2020

| **Deaths** | | | | |
| --- | --- | --- | --- | --- |
| **Year** | **Medical**  **Facility** | **Nursing Home/Long-term**  **Care Facility** | **Hospices** | **Home** |
| 1999 | 6667 | 721 | N/A | 905 |
| 2000 | 6342 | 648 | N/A | 826 |
| 2001 | 5809 | 652 | N/A | 840 |
| 2002 | 5713 | 630 | N/A | 774 |
| 2003 | 5380 | 626 | N/A | 859 |
| 2004 | 4867 | 563 | N/A | 823 |
| 2005 | 4802 | 559 | 33 | 769 |
| 2006 | 4588 | 545 | 31 | 716 |
| 2007 | 4492 | 492 | 49 | 721 |
| 2008 | 4433 | 435 | 68 | 703 |
| 2009 | 4359 | 468 | 75 | 699 |
| 2010 | 4526 | 479 | 81 | 750 |
| 2011 | 4643 | 505 | 104 | 798 |
| 2012 | 4683 | 445 | 122 | 817 |
| 2013 | 4750 | 470 | 148 | 957 |
| 2014 | 4967 | 458 | 130 | 962 |
| 2015 | 5263 | 503 | 176 | 1040 |
| 2016 | 5611 | 521 | 170 | 1091 |
| 2017 | 5922 | 527 | 221 | 1175 |
| 2018 | 6149 | 561 | 239 | 1316 |
| 2019 | 6485 | 563 | 248 | 1344 |
| 2020 | 7148 | 534 | 247 | 1848 |
| **Total** | 117599 | 11905 | 2153 | 20733 |

N/A = not available (unreliable or suppressed)
