# Supplementary material for: Trends, gender, and racial disparities in patients with mortality due to paroxysmal tachycardia: A nationwide analysis from 1999–2020
Source: PLoS One. 2025 Feb 4;20(2):e0314715. doi: 10.1371/journal.pone.0314715 (PMC11793763; doi:10.1371/journal.pone.0314715)
Supplement: S3 Table — APC = Annual percent change; NH = non-Hispanic. (DOCX) [file pone.0314715.s003.docx]

**S3 Table.** Annual percent change (APC) of Paroxysmal Tachycardia–related Age-Adjusted Mortality Rates per 100,000 in Adults in the United States, 1999 to 2020

| Year Interval | APC (95% CI) |
| --- | --- |
| Overall | |
| 1999-2007 | -6.24 (-6.82 to -5.78) |
| 2007-2014 | -0.22 (-1.34 to 0.85) |
| 2014- 2020 | 4.33 (3.53 to 5.56) |
| Men | |
| 1999-2007 | -6.26 (-7.15 to -5.65) |
| 2007-2013 | -0.38 (-2.72 to 1.38) |
| 2013-2020 | 3.95 (3.18 to 5.46) |
| Women | |
| 1999-2007 | -6.03 (-8.01 to -4.50) |
| 2007-2012 | -1.56 (-7.52 to 0.14) |
| 2012-2018 | 1.64 (-1.50 to 3.27) |
| 2018-2020 | 7.55 (3.18 to 10.09) |
| Young Adults (25-44 years) | |
| 1999-2001 | -17.28 (-24.49 to -4.77) |
| 2001-2013 | 0.22 (-1.81 to 2.44) |
| 2013-2020 | 6.60 (3.53 to 16.37) |
| Middle Aged Adults (46-64 years) | |
| 1999-2005 | -7.27 (-11.06 to -5.66) |
| 2005-2012 | -1.60 (-3.51 to 1.86) |
| 2012-2020 | 6.40 (5.35 to 7.98) |
| Older Adults (65 years and above) | |
| 1999-2007 | -6.09 (-6.78 to -5.61) |
| 2007-2014 | -0.80 (-2.43 to 0.50) |
| 2014-2020 | 3.70 (2.66 to 5.64) |
| NH American Indian or Alaska Native | |
| 1999-2014 | -2.24 (-17.81 to 1.37) |
| 2014-2020 | 8.40 (0.53 to 33.72) |
| NH White | |
| 1999-2006 | -6.05 (-7.57 to -5.39) |
| 2006-2010 | -2.76 (-5.07 to 0.71) |
| 2010-2018 | 2.05 (0.87 to 2.89) |
| 2018-2020 | 7.69 (4.42 to 9.67) |
| NH Black or African American | |
| 1999-2008 | -5.55 (-8.68 to -4.15) |
| 2008-2016 | 1.00 (-4.60 to 2.87) |
| 2016-2020 | 6.52 (3.11 to 12.90) |
| Hispanic or Latino | |
| 1999-2010 | -7.01 (-9.12 to -5.04) |
| 2010-2020 | 5.21 (3.45 to 8.09) |
| NH Asian or Pacific Islander | |
| 1999-2002 | -3.31 (-9.11 to 5.52) |
| 2002-2006 | -13.31 (-19.27 to 6.23) |
| 2006-2009 | 10.11 (-4.42 to 15.55) |
| 2009-2014 | -4.36 (-9.89 to 2.62) |
| 2014-2020 | 6.45 (4.48 to 10.44) |
| Non-metropolitan areas | |
| 1999-2006 | -5.51 (-7.45 to -4.37) |
| 2006-2012 | -1.56 (-6.40 to 0.30) |
| 2012-2018 | 2.61 (-1.35 to 4.14) |
| 2018-2020 | 8.96 (4.35 to 11.92) |
| Metropolitan area | |
| 1999-2007 | -6.31 (-6.89 to -5.84) |
| 2007-2014 | -0.28 (-1.23 to 0.69) |
| 2014-2020 | 4.37 (3.63 to 5.47) |
| Northeast | |
| 1999-2007 | -7.48 (-8.42 to -6.89) |
| 2007-2018 | 1.01 (0.10 to 1.75) |
| 2018-2020 | 11.50 (5.78 to 14.42) |
| Midwest | |
| 1999-2009 | -5.85 (-6.68 to -5.18) |
| 2009-2020 | 2.91 (2.18 to 3.83) |
| South | |
| 1999-2005 | -6.73 (-8.19 to -5.93) |
| 2005-2012 | -2.55 (-3.89 to -0.79) |
| 2012-2020 | 3.45 (2.73 to 4.47) |
| West | |
| 1999-2006 | -5.21 (-9.47 to -3.65) |
| 2006-2013 | -1.41 (-3.91 to 3.97) |
| 2013-2020 | 5.20 (3.29 to 10.88) |

APC = Annual percent change; NH = non-Hispanic.
