# Supplementary material for: Trends, gender, and racial disparities in patients with mortality due to paroxysmal tachycardia: A nationwide analysis from 1999–2020
Source: PLoS One. 2025 Feb 4;20(2):e0314715. doi: 10.1371/journal.pone.0314715 (PMC11793763; doi:10.1371/journal.pone.0314715)
Supplement: S4 Table — (DOCX) [file pone.0314715.s004.docx]

**S4 Table.** Overall and Sex‐Stratified Paroxysmal Tachycardia–related Age-Adjusted Mortality Rates per 100,000 in Adults in the United States from 1999 to 2020

| Age-Adjusted Rate (95% CI) | | | |
| --- | --- | --- | --- |
| Year | **Men** | **Women** | **Overall** |
| 1999 | 6.8 (6.6-7.0) | 3.4 (3.2-3.5) | 4.8 (4.7-4.9) |
| 2000 | 6.4 (6.2-6.5) | 3.1 (3.0-3.2) | 4.4 (4.3-4.5) |
| 2001 | 5.8 (5.7-6.0) | 2.9 (2.8-3.0) | 4.1 (4.0-4.2) |
| 2002 | 5.5 (5.4-5.7) | 2.8 (2.7-2.9) | 3.9 (3.8-4.0) |
| 2003 | 5.3 (5.2-5.5) | 2.6 (2.5-2.7) | 3.7 (3.6-3.8) |
| 2004 | 4.8 (4.6-4.9) | 2.4 (2.3-2.5) | 3.4 (3.3-3.4) |
| 2005 | 4.6 (4.5-4.8) | 2.3 (2.2-2.4) | 3.2 (3.2-3.3) |
| 2006 | 4.3 (4.1-4.4) | 2.2 (2.1-2.2) | 3.0 (3.0-3.1) |
| 2007 | 4.1 (4.0-4.2) | 2.0 (2.0-2.1) | 2.9 (2.8-3.0) |
| 2008 | 4.0 (3.9-4.1) | 2.0 (1.9-2.1) | 2.8 (2.8-2.9) |
| 2009 | 3.9 (3.8-4.1) | 2.0 (1.9-2.0) | 2.8 (2.7-2.9) |
| 2010 | 4.0 (3.9-4.1) | 1.9 (1.9-2.0) | 2.8 (2.7-2.9) |
| 2011 | 4.0 (3.9-4.1) | 1.9 (1.8-2.0) | 2.8 (2.8-2.9) |
| 2012 | 3.9 (3.8-4.0) | 1.9 (1.8-2.0) | 2.8 (2.7-2.8) |
| 2013 | 4.0 (3.9-4.2) | 1.9 (1.8-1.9) | 2.8 (2.7-2.9) |
| 2014 | 4.1 (3.9-4.2) | 1.9 (1.8-2.0) | 2.8 (2.8-2.9) |
| 2015 | 4.2 (4.1-4.4) | 2.0 (1.9-2.1) | 2.9 (2.9-3.0) |
| 2016 | 4.5 (4.4-4.6) | 2.0 (1.9-2.0) | 3.1 (3.0-3.1) |
| 2017 | 4.6 (4.5-4.8) | 2.0 (1.9-2.1) | 3.1 (3.1-3.2) |
| 2018 | 4.7 (4.6-4.8) | 2.1 (2.0-2.2) | 3.3 (3.2-3.3) |
| 2019 | 4.8 (4.7-4.9) | 2.2 (2.1-2.3) | 3.3 (3.3-3.4) |
| 2020 | 5.3 (5.2-5.5) | 2.4 (2.4-2.5) | 3.7 (3.6-3.8) |
| Overall | 4.7 (4.6-4.7) | 2.2 (2.2-2.2) | 3.3 (3.2-3.3) |
