# Supplementary material for: Trends, gender, and racial disparities in patients with mortality due to paroxysmal tachycardia: A nationwide analysis from 1999–2020
Source: PLoS One. 2025 Feb 4;20(2):e0314715. doi: 10.1371/journal.pone.0314715 (PMC11793763; doi:10.1371/journal.pone.0314715)
Supplement: S5 Table — Young Adult = 25–44 years; Middle Aged Adults = 45–64 years; Older Adults = 65 years and above. (DOCX) [file pone.0314715.s005.docx]

**S5 Table.** Paroxysmal Tachycardia-related Mortality, Stratified by Age groups in Adults in the United States, 1999 to 2020.

|  | **Deaths** | | | **Age-Adjusted Rate (95% CI)** | | |
| --- | --- | --- | --- | --- | --- | --- |
| **Year** | **Young Adults** | **Middle Aged Adults** | **Older Adults** | **Young Adults** | **Middle Aged Adults** | **Older Adults** |
| 1999 | 203 | 1463 | 6721 | 0.3 (0.2-0.3) | 2.4 (2.3-2.5) | 19.4 (19.0-18.6) |
| 2000 | 175 | 1292 | 6466 | 0.2 (0.2-0.2) | 2.1 (2.0-2.2) | 18.6 (18.1-17.6) |
| 2001 | 183 | 1296 | 5937 | 0.2 (0.2-0.2) | 2.0 (1.9-2.1) | 16.8 (16.4-16.0) |
| 2002 | 146 | 1301 | 5780 | 0.2 (0.1-0.2) | 1.9 (1.8-2.1) | 16.2 (15.8-15.4) |
| 2003 | 148 | 1249 | 5585 | 0.2 (0.1-0.2) | 1.8 (1.7-1.9) | 15.5 (15.1-14.7) |
| 2004 | 180 | 1168 | 5050 | 0.2 (0.2-0.2) | 1.6 (1.5-1.7) | 13.9 (13.5-13.1) |
| 2005 | 155 | 1138 | 4990 | 0.2 (0.2-0.2) | 1.5 (1.4-1.6) | 13.5 (13.1-12.7) |
| 2006 | 131 | 1127 | 4730 | 0.2 (0.1-0.2) | 1.5 (1.4-1.6) | 12.6 (12.2-11.8) |
| 2007 | 174 | 1097 | 4588 | 0.2 (0.2-0.2) | 1.4 (1.3-1.5) | 12.0 (11.6-11.2) |
| 2008 | 131 | 1150 | 4530 | 0.2 (0.1-0.2) | 1.4 (1.4-1.5) | 11.6 (11.2-10.8) |
| 2009 | 154 | 1138 | 4545 | 0.2 (0.2-0.2) | 1.4 (1.3-1.4) | 11.4 (11.1-10.8) |
| 2010 | 145 | 1146 | 4653 | 0.2 (0.2-0.2) | 1.4 (1.3-1.4) | 11.5 (11.2-10.9) |
| 2011 | 139 | 1264 | 4758 | 0.2 (0.2-0.2) | 1.4 (1.4-1.5) | 11.4 (11.1-10.8) |
| 2012 | 157 | 1211 | 4815 | 0.2 (0.2-0.2) | 1.3 (1.3-1.4) | 11.2 (10.9-10.6) |
| 2013 | 160 | 1340 | 4951 | 0.2 (0.2-0.2) | 1.5 (1.4-1.6) | 11.2 (10.9-10.6) |
| 2014 | 164 | 1333 | 5153 | 0.2 (0.2-0.2) | 1.5 (1.4-1.6) | 11.4 (11.0-10.6) |
| 2015 | 178 | 1505 | 5435 | 0.2 (0.2-0.2) | 1.6 (1.5-1.7) | 11.7 (11.4-11.1) |
| 2016 | 198 | 1591 | 5721 | 0.3 (0.2-0.3) | 1.7 (1.6-1.8) | 12.0 (11.7-11.4) |
| 2017 | 201 | 1674 | 6111 | 0.2 (0.2-0.2) | 1.8 (1.7-1.9) | 12.4 (12.1-11.8) |
| 2018 | 227 | 1748 | 6434 | 0.3 (0.2-0.3) | 1.9 (1.8-2.0) | 12.7 (12.4-12.1) |
| 2019 | 255 | 1845 | 6679 | 0.3 (0.3-0.4) | 2.0 (1.9-2.1) | 12.9 (12.6-12.3) |
| 2020 | 248 | 2118 | 7642 | 0.3 (0.2-0.3) | 2.3 (2.2-2.4) | 14.3 (14.0-13.7) |
| **Overall** | 3852 | 30194 | 121274 | 0.2 (0.2-0.2) | 1.7 (1.6-1.7) | 13.3 (13.2-13.1) |

Young Adult = 25-44 years; Middle Aged Adults = 45-64 years; Older Adults = 65 years and above
