# Supplementary material for: Trends, gender, and racial disparities in patients with mortality due to paroxysmal tachycardia: A nationwide analysis from 1999–2020
Source: PLoS One. 2025 Feb 4;20(2):e0314715. doi: 10.1371/journal.pone.0314715 (PMC11793763; doi:10.1371/journal.pone.0314715)
Supplement: S6 Table — NH = non-Hispanic. (DOCX) [file pone.0314715.s006.docx]

**S6 Table.** Paroxysmal Tachycardia-related Age-Adjusted Mortality Rates per 100,000 stratified by Race in Adults in the United States from 1999 to 2020

| Age-Adjusted Rate (95% CI) | | | | | |
| --- | --- | --- | --- | --- | --- |
| Year | **NH White** | **NH Black or African American** | **NH American Indian or Alaska Native** | **Hispanic or Latino** | **NH Asian or Pacific Islander** |
| 1999 | 4.8 (4.6-4.9) | 5.8 (5.4-6.2) | 3.4 (2.2-5.0) | 3.2 (2.8-3.6) | 2.8 (2.3-3.4) |
| 2000 | 4.5 (4.4-4.6) | 5.0 (4.7-5.4) | 3.8 (2.5-5.5) | 3.3 (2.9-3.7) | 2.5 (2.0-3.0) |
| 2001 | 4.1 (4.0-4.2) | 5.3 (4.9-5.6) | 3.1 (2.0-4.5) | 2.9 (2.5-3.2) | 2.5 (2.0-3.0) |
| 2002 | 4.0 (3.9-4.1) | 4.7 (4.3-5.0) | 2.6 (1.6-4.0) | 2.4 (2.1-2.7) | 2.5 (2.1-3.0) |
| 2003 | 3.8 (3.7-3.9) | 4.2 (3.9-4.6) | 4.4 (3.0-6.2) | 2.4 (2.1-2.7) | 2.1 (1.7-2.5) |
| 2004 | 3.4 (3.3-3.5) | 4.2 (3.9-4.5) | 4.1 (2.8-5.9) | 2.2 (1.9-2.5) | 1.8 (1.5-2.2) |
| 2005 | 3.3 (3.2-3.3) | 3.8 (3.5-4.1) | 3.3 (2.2-4.9) | 2.4 (2.1-2.7) | 1.7 (1.4-2.1) |
| 2006 | 3.1 (3.0-3.1) | 3.9 (3.6-4.2) | 4.0 (2.8-5.6) | 1.9 (1.7-2.2) | 1.4 (1.1-1.8) |
| 2007 | 3.0 (2.9-3.0) | 3.6 (3.4-3.9) | 2.8 (1.7-4.1) | 1.7 (1.5-1.9) | 1.4 (1.2-1.8) |
| 2008 | 2.9 (2.8-3.0) | 3.4 (3.1-3.7) | 2.2 (1.4-3.4) | 1.7 (1.5-1.9) | 1.7 (1.4-2.0) |
| 2009 | 2.8 (2.8-2.9) | 3.3 (3.1-3.6) | 2.9 (1.9-4.2) | 1.7 (1.5-1.9) | 1.8 (1.5-2.1) |
| 2010 | 2.8 (2.7-2.9) | 3.5 (3.2-3.8) | 3.9 (2.7-5.3) | 1.6 (1.4-1.8) | 1.7 (1.4-2.0) |
| 2011 | 2.9 (2.8-3.0) | 3.5 (3.2-3.7) | 2.3 (1.5-3.5) | 1.7 (1.5-1.9) | 1.8 (1.5-2.0) |
| 2012 | 2.8 (2.8-2.9) | 3.3 (3.1-3.6) | 3.0 (2.1-4.3) | 1.6 (1.4-1.8) | 1.7 (1.4-2.0) |
| 2013 | 2.9 (2.8-3.0) | 3.5 (3.2-3.7) | 2.3 (1.5-3.2) | 1.7 (1.5-1.9) | 1.5 (1.2-1.7) |
| 2014 | 2.9 (2.8-3.0) | 3.6 (3.4-3.9) | 2.3 (1.6-3.3) | 1.7 (1.5-1.9) | 1.4 (1.2-1.7) |
| 2015 | 3.1 (3.0-3.1) | 3.8 (3.6-4.1) | 2.9 (2.1-4.1) | 1.6 (1.4-1.8) | 1.6 (1.3-1.8) |
| 2016 | 3.1 (3.1-3.2) | 3.5 (3.2-3.7) | 3.3 (2.4-4.4) | 2.2 (2.0-2.4) | 1.7 (1.4-1.9) |
| 2017 | 3.2 (3.2-3.3) | 3.9 (3.6-4.1) | 4.5 (3.5-5.8) | 2.0 (1.8-2.2) | 1.7 (1.5-1.9) |
| 2018 | 3.3 (3.3-3.4) | 4.1 (3.8-4.3) | 3.4 (2.6-4.5) | 2.2 (2.0-2.4) | 1.9 (1.6-2.1) |
| 2019 | 3.4 (3.3-3.5) | 4.2 (3.9-4.4) | 2.9 (2.1-3.8) | 2.2 (2.0-2.4) | 2.1 (1.9-2.4) |
| 2020 | 3.8 (3.7-3.9) | 4.8 (4.5-5.1) | 4.8 (3.8-5.9) | 2.7 (2.5-2.9) | 2.1 (1.9-2.4) |
| Overall | 3.3 (3.3-3.4) | 4.0 (3.9-4.1) | 3.3 (3.0-3.5) | 2.1 (2.1-2.2) | 1.9 (1.8-1.9) |

NH=non-Hispanic
