# Supplementary material for: Trends, gender, and racial disparities in patients with mortality due to paroxysmal tachycardia: A nationwide analysis from 1999–2020
Source: PLoS One. 2025 Feb 4;20(2):e0314715. doi: 10.1371/journal.pone.0314715 (PMC11793763; doi:10.1371/journal.pone.0314715)
Supplement: S7 Table — (DOCX) [file pone.0314715.s007.docx]

**S7 Table.** Paroxysmal Tachycardia–related Age-Adjusted Mortality Rates per 100,000, Stratified by State in Adults in the United States, 1999 to 2020.

| State | Age-Adjusted Rate (95% CI) |
| --- | --- |
| Alabama | 3.4 (3.3-3.6) |
| Alaska | 3.0 (2.6-3.5) |
| Arizona | 2.7 (2.6-2.8) |
| Arkansas | 3.7 (3.5-3.9) |
| California | 3.0 (3.0-3.0) |
| Colorado | 3.5 (3.3-3.6) |
| Connecticut | 3.0 (2.9-3.2) |
| Delaware | 3.8 (3.4-4.1) |
| District of Columbia | 2.9 (2.5-3.3) |
| Florida | 2.7 (2.6-2.7) |
| Georgia | 2.9 (2.8-3.0) |
| Hawaii | 3.7 (3.4-3.9) |
| Idaho | 3.0 (2.8-3.2) |
| Illinois | 2.8 (2.8-2.9) |
| Indiana | 4.4 (4.3-4.5) |
| Iowa | 3.0 (2.9-3.2) |
| Kansas | 3.4 (3.2-3.5) |
| Kentucky | 3.8 (3.7-4.0) |
| Louisiana | 2.5 (2.4-2.6) |
| Maine | 3.2 (3.0-3.5) |
| Maryland | 3.4 (3.2-3.5) |
| Massachusetts | 2.7 (2.6-2.8) |
| Michigan | 2.8 (2.7-2.9) |
| Minnesota | 2.7 (2.6-2.9) |
| Mississippi | 3.2 (3.1-3.4) |
| Missouri | 4.0 (3.9-4.2) |
| Montana | 2.8 (2.6-3.1) |
| Nebraska | 3.2 (3.0-3.4) |
| Nevada | 2.8 (2.6-3.0) |
| New Hampshire | 2.8 (2.6-3.0) |
| New Jersey | 3.4 (3.3-3.5) |
| New Mexico | 2.6 (2.4-2.8) |
| New York | 2.2 (2.1-2.2) |
| North Carolina | 4.0 (3.9-4.1) |
| North Dakota | 3.3 (3.0-3.6) |
| Ohio | 4.3 (4.2-4.4) |
| Oklahoma | 3.1 (3.0-3.3) |
| Oregon | 3.5 (3.4-3.6) |
| Pennsylvania | 4.1 (4.0-4.2) |
| Rhode Island | 3.4 (3.1-3.6) |
| South Carolina | 4.7 (4.5-4.8) |
| South Dakota | 2.9 (2.7-3.2) |
| Tennessee | 4.5 (4.3-4.6) |
| Texas | 3.6 (3.6-3.7) |
| Utah | 2.8 (2.6-3.0) |
| Vermont | 3.6 (3.2-3.9) |
| Virginia | 3.1 (3.0-3.3) |
| Washington | 3.6 (3.4-3.7) |
| West Virginia | 4.7 (4.5-4.9) |
| Wisconsin | 3.0 (2.9-3.1) |
| Wyoming | 3.4 (3.0-3.8) |
| Overall | 3.3 (3.2-3.3) |
