# Supplementary material for: Trends, gender, and racial disparities in patients with mortality due to paroxysmal tachycardia: A nationwide analysis from 1999–2020
Source: PLoS One. 2025 Feb 4;20(2):e0314715. doi: 10.1371/journal.pone.0314715 (PMC11793763; doi:10.1371/journal.pone.0314715)
Supplement: S8 Table — (DOCX) [file pone.0314715.s008.docx]

**S8 Table.** Paroxysmal Tachycardia related Age-Adjusted Mortality Rate per 100,000 Stratified by Census Region in Adults in the United States 1999-2020.

| Census Region | Year | Age-Adjusted Rate (95% CI) |
| --- | --- | --- |
| Northeast | 1999 | 4.6 (4.4-4.8) |
| Northeast | 2000 | 4.4 (4.2-4.6) |
| Northeast | 2001 | 4.0 (3.8-4.2) |
| Northeast | 2002 | 3.7 (3.5-3.9) |
| Northeast | 2003 | 3.5 (3.3-3.7) |
| Northeast | 2004 | 3.1 (2.9-3.3) |
| Northeast | 2005 | 3.0 (2.8-3.2) |
| Northeast | 2006 | 2.7 (2.6-2.9) |
| Northeast | 2007 | 2.6 (2.5-2.8) |
| Northeast | 2008 | 2.5 (2.3-2.6) |
| Northeast | 2009 | 2.5 (2.3-2.6) |
| Northeast | 2010 | 2.5 (2.4-2.7) |
| Northeast | 2011 | 2.7 (2.5-2.9) |
| Northeast | 2012 | 2.6 (2.4-2.7) |
| Northeast | 2013 | 2.6 (2.4-2.7) |
| Northeast | 2014 | 2.7 (2.5-2.8) |
| Northeast | 2015 | 2.7 (2.6-2.9) |
| Northeast | 2016 | 2.7 (2.6-2.9) |
| Northeast | 2017 | 2.8 (2.7-3.0) |
| Northeast | 2018 | 2.9 (2.8-3.1) |
| Northeast | 2019 | 3.1 (2.9-3.2) |
| Northeast | 2020 | 3.5 (3.3-3.6) |
| Northeast | **Overall** | 3.0 (3.0-3.1) |
| Midwest | 1999 | 5 (4.8-5.2) |
| Midwest | 2000 | 4.5 (4.3-4.7) |
| Midwest | 2001 | 4.3 (4.1-4.5) |
| Midwest | 2002 | 4.1 (3.9-4.3) |
| Midwest | 2003 | 3.8 (3.7-4.0) |
| Midwest | 2004 | 3.5 (3.3-3.7) |
| Midwest | 2005 | 3.4 (3.2-3.6) |
| Midwest | 2006 | 3.3 (3.1-3.4) |
| Midwest | 2007 | 3.0 (2.8-3.2) |
| Midwest | 2008 | 2.8 (2.6-3.0) |
| Midwest | 2009 | 2.9 (2.7-3.0) |
| Midwest | 2010 | 2.7 (2.6-2.9) |
| Midwest | 2011 | 2.8 (2.7-3.0) |
| Midwest | 2012 | 2.9 (2.7-3.0) |
| Midwest | 2013 | 3.1 (2.9-3.2) |
| Midwest | 2014 | 2.9 (2.8-3.1) |
| Midwest | 2015 | 3.2 (3.0-3.3) |
| Midwest | 2016 | 3.1 (2.9-3.3) |
| Midwest | 2017 | 3.4 (3.2-3.5) |
| Midwest | 2018 | 3.3 (3.2-3.5) |
| Midwest | 2019 | 3.5 (3.4-3.7) |
| Midwest | 2020 | 3.9 (3.8-4.1) |
| Midwest | **Overall** | 3.4 (3.4-3.4) |
| South | 1999 | 5 (4.8-5.2) |
| South | 2000 | 4.7 (4.5-4.8) |
| South | 2001 | 4.3 (4.1-4.5) |
| South | 2002 | 4.2 (4.0-4.4) |
| South | 2003 | 3.9 (3.8-4.1) |
| South | 2004 | 3.5 (3.4-3.7) |
| South | 2005 | 3.3 (3.1-3.4) |
| South | 2006 | 3.2 (3.0-3.3) |
| South | 2007 | 3.1 (3.0-3.3) |
| South | 2008 | 3.1 (3.0-3.2) |
| South | 2009 | 2.9 (2.8-3.0) |
| South | 2010 | 2.9 (2.8-3.1) |
| South | 2011 | 2.9 (2.8-3.0) |
| South | 2012 | 2.8 (2.7-2.9) |
| South | 2013 | 2.9 (2.8-3.0) |
| South | 2014 | 2.9 (2.8-3.1) |
| South | 2015 | 3.0 (2.9-3.1) |
| South | 2016 | 3.2 (3.1-3.3) |
| South | 2017 | 3.3 (3.2-3.4) |
| South | 2018 | 3.4 (3.2-3.5) |
| South | 2019 | 3.4 (3.3-3.5) |
| South | 2020 | 3.7 (3.6-3.8) |
| South | **Overall** | 3.4 (3.4-3.4) |
| West | 1999 | 4.1 (3.8-4.3) |
| West | 2000 | 4.0 (3.8-4.2) |
| West | 2001 | 3.6 (3.4-3.8) |
| West | 2002 | 3.4 (3.3-3.6) |
| West | 2003 | 3.4 (3.2-3.6) |
| West | 2004 | 3.1 (2.9-3.3) |
| West | 2005 | 3.2 (3.0-3.3) |
| West | 2006 | 2.7 (2.6-2.9) |
| West | 2007 | 2.7 (2.6-2.9) |
| West | 2008 | 2.7 (2.6-2.9) |
| West | 2009 | 2.7 (2.5-2.9) |
| West | 2010 | 2.8 (2.6-3.0) |
| West | 2011 | 2.7 (2.6-2.9) |
| West | 2012 | 2.6 (2.5-2.8) |
| West | 2013 | 2.5 (2.4-2.7) |
| West | 2014 | 2.7 (2.5-2.8) |
| West | 2015 | 2.8 (2.6-2.9) |
| West | 2016 | 3.1 (2.9-3.2) |
| West | 2017 | 3.0 (2.9-3.2) |
| West | 2018 | 3.3 (3.1-3.4) |
| West | 2019 | 3.3 (3.2-3.5) |
| West | 2020 | 3.8 (3.6-3.9) |
| West | **Overall** | 3.1 (3.0-3.1) |
