# Supplementary material for: Trends, gender, and racial disparities in patients with mortality due to paroxysmal tachycardia: A nationwide analysis from 1999–2020
Source: PLoS One. 2025 Feb 4;20(2):e0314715. doi: 10.1371/journal.pone.0314715 (PMC11793763; doi:10.1371/journal.pone.0314715)
Supplement: S9 Table — (DOCX) [file pone.0314715.s009.docx]

**S9 Table.** Overall Paroxysmal Tachycardia–related Age-Adjusted Mortality Rates per 100,000 in Adults in the Metropolitan and Non-metropolitan areas in the United States, 1999 to 2020

| Age-Adjusted Rate (95% CI) | | |
| --- | --- | --- |
| Year | **Metropolitan** | **Nonmetropolitan** |
| 1999 | 4.68 (4.57-4.79) | 5.1 (4.8-5.3) |
| 2000 | 4.42 (4.31-4.53) | 4.6 (4.3-4.8) |
| 2001 | 4.02 (3.92-4.12) | 4.4 (4.1-4.6) |
| 2002 | 3.89 (3.79-3.99) | 4.1 (3.8-4.3) |
| 2003 | 3.63 (3.54-3.73) | 4.1 (3.8-4.3) |
| 2004 | 3.26 (3.17-3.35) | 3.7 (3.5-3.9) |
| 2005 | 3.16 (3.07-3.24) | 3.6 (3.4-3.8) |
| 2006 | 2.96 (2.88-3.04) | 3.4 (3.2-3.6) |
| 2007 | 2.84 (2.76-2.92) | 3.3 (3.1-3.5) |
| 2008 | 2.75 (2.67-2.83) | 3.2 (3-3.4) |
| 2009 | 2.73 (2.65-2.81) | 3.1 (2.9-3.3) |
| 2010 | 2.71 (2.63-2.78) | 3.2 (3-3.4) |
| 2011 | 2.77 (2.70-2.85) | 3.1 (2.9-3.2) |
| 2012 | 2.69 (2.61-2.76) | 3 (2.9-3.2) |
| 2013 | 2.74 (2.66-2.81) | 3.2 (3-3.3) |
| 2014 | 2.74 (2.67-2.82) | 3.2 (3.1-3.4) |
| 2015 | 2.88 (2.81-2.96) | 3.3 (3.2-3.5) |
| 2016 | 2.97 (2.90-3.05) | 3.4 (3.2-3.5) |
| 2017 | 3.07 (2.99-3.14) | 3.5 (3.3-3.7) |
| 2018 | 3.19 (3.12-3.27) | 3.6 (3.4-3.8) |
| 2019 | 3.26 (3.18-3.33) | 3.7 (3.6-3.9) |
| 2020 | 3.61 (3.53-3.69) | 4.3 (4.1-4.5) |
| Overall | 3.18 (3.16-3.19) | 3.6 (3.6-3.6) |
