# Supplementary figures and images for: Trends, gender, and racial disparities in patients with mortality due to paroxysmal tachycardia: A nationwide analysis from 1999–2020
Source: PLoS One. 2025 Feb 4;20(2):e0314715. doi: 10.1371/journal.pone.0314715 (PMC11793763; doi:10.1371/journal.pone.0314715)

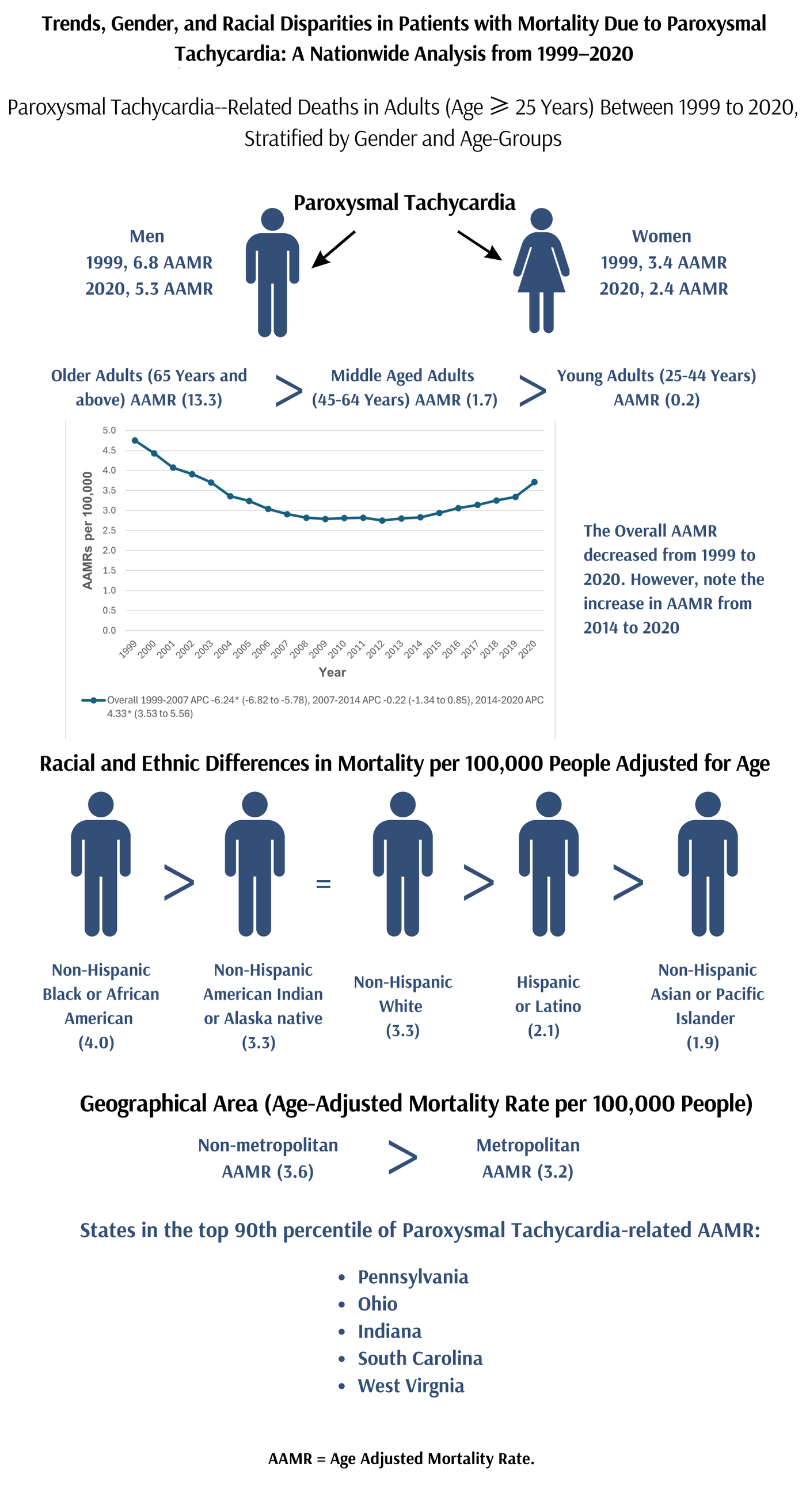

Supplement: S1 Graphical abstract — (TIF) [file pone.0314715.s010.tif]
